# Sex Differences in Glioblastoma—Findings from the Swedish National Quality Registry for Primary Brain Tumors between 1999–2018

**DOI:** 10.3390/jcm11030486

**Published:** 2022-01-18

**Authors:** Björn Tavelin, Annika Malmström

**Affiliations:** 1 Department of Radiation Sciences, Oncology, Umeå University, 90187 Umeå, Sweden; bjorn.tavelin@umu.se; 2 Department of Biomedical and Clinical Sciences, Linköping University, 58185 Linköping, Sweden; 3 Department of Advanced Home Care, Linköping University, 58185 Linköping, Sweden

**Keywords:** glioblastoma, population-based cohort, sex differences, clinical factors, survival

## Abstract

Sex disparities in glioblastoma (GBM) have received increasing attention. Sex-related differences for several molecular markers have been reported, which could impact on clinical factors and outcomes. We therefore analyzed data on all patients with GBM reported to the Swedish National Quality Registry for Primary Brain Tumors, according to sex, with a focus on prognostic factors and survival. All glioma patients registered during 20 years, from 1 January 1999 until 31 December 2018, with SNOMED codes 94403, 94413, and 94423, were analyzed. Chi^2^-test, log-rank test, and Kaplan–Meier analyses were performed. We identified 5243 patients, of which 2083 were females and 3160 males, resulting in a ratio of 1:1.5. We found sex related differences, with women having diagnostic surgery at a significantly higher age (*p* = 0.001). Women were also reported to have a worse preoperative performance status (PPS) (<0.001). There was no gender difference for the type of surgery performed. For women with radical surgery, overall survival was slightly better than for men (*p* = 0.045). The time period did not influence survival, neither for 1999–2005 nor 2006–2018, after temozolomide treatment was introduced (*p* = 0.35 and 0.10, respectively). In the multivariate analysis including sex, age, surgery, and PPS, a survival advantage was noted for women, but this was not clinically relevant (HR = 0.92, *p* = 0.006). For patients with GBM; sex-related differences in clinical factors could be identified in a population-based cohort. In this dataset, for survival, the only advantage noted was for women who had undergone radical surgery, although this was clinically almost negligible.

## 1. Introduction

Glioblastoma (GBM) is the most common and aggressive glioma, with the median survival for unselected, population-based cohorts reported to be between 9–11 months [1,2]. For those with good prognostic factors, allowing for extensive, multimodal treatment with surgery, radiation, chemotherapy, and tumor treating fields, the median survival can be prolonged to nearly 21 months [3]. A well-known fact is that men are affected more often than women, with the ratio between men and women reported in the literature as being 1.4–1.6:1 [4,5]. This relationship has received increasing attention in recent years, with researchers investigating the role of molecular patterns related to sex. As a consequence, this has led to an increased understanding of the basic sex differences, identified for mutations [6] and methylation profiles [7], followed by transcriptomes [8] and tumor metabolism [9]. In addition, an influence in the proportion of patients being MGMT promoter methylated and response to alkylating agent treatment in a sex specific manner have been identified [10]. Whether survival is affected by these disparities is debated [8,11,12,13].

In Sweden, the National Quality Registry for Primary Brain Tumors (SQRBT) was launched in 1999, covering all primary intracranial tumor patients, 18 years and older, in the six regions of Sweden. The collected data include clinical factors, such as age, sex, preoperative performance status (PPS), type of surgery, pathology, and survival, as well as lead times. In later years, oncological treatment, patient reported outcome measures (PROM), and patient reported experience measures (PREM) have also been collected. Since 2018, molecular markers, IDH1/2 mutations, codeletion of 1p/19q, and MGMT promoter methylation status have been reported as well. As these data require longer follow-up, they are not reported here.

Since 1999, there have been several updates on the pathology of tumors of the central nervous system. In 2007, a minor revision in the pathology guidelines was published [14]. A change with a larger impact occurred in 2016, when the WHO guidelines for primary brain tumors were updated to include molecular markers [5]. This led to many tumors previously diagnosed as grade 3 glioma being reclassified as IDH wildtype (IDHwt) GBM, for example in the NOA-04 and CATNON studies, where around 30% were found to be IDHwt tumors [15,16]. Approximately 10% of GBMs have been identified as IDH mutated (IDHmut), these previously being secondary GBMs [5], but according to the latest guidelines they are now astrocytoma grade 4 [17,18].

Sex disparities, some related to the increasing number of molecular differences between males and females that have been identified in later years in GBM, could potentially affect the outcome in a population-based cohort. We therefore here report on the sex specific analyses in relation to the clinical and prognostic factors for all Swedish patients diagnosed with GBM and reported in the SQRBT.

## 2. Materials and Methods

Patients registered in the SQRBT between 1 January 1999 and 31 December 2018 were identified. Due to the above-mentioned change in the WHO tumor classification since the start of the registry, we focused on those reported as GBM, giant cell GBM, or gliosarcoma, with SNOMED codes 94403, 94413, and 94423, respectively, as in this cohort only a minority of the GBMs are expected to be IDHmut tumors. This is also in line with previous publications from the National Cancer Database; the Central Brain Tumor Registry of the United States; and Surveillance, Epidemiology and End Results (SEER) databases [2,19].

Registered prognostic and clinical factors were retrieved and analyzed for the whole cohort, subdivided by sex. We focused on patient related factors, including age at diagnosis, WHO PPS, and treatment related factors, such as type of surgery. We investigated the lead times according to sex. We subdivided the cohort into two different time periods to check for any changes in sex related survival over time. The cut-off chosen was 1999–2005, being before the introduction of combined radio-chemotherapy with temozolomide, and 2006–2018, when it had been introduced into clinical practice. Comparison of groups were done by Chi square test and survival was calculated with the log rank and the Kaplan–Meier method. Survival was also calculated using multivariate analysis, taking the prognostic factors PPS, age, and type of surgery into account.

This study was approved by the Ethical Review Authority, Umeå, Sweden, numbers 2014-95-31 and 2020-06886.

All of the statistical analyses were performed with IBM SPSS Statistics for Windows, Version 26.0. Armonk, NY, USA.

## 3. Results

**Figure 1:**
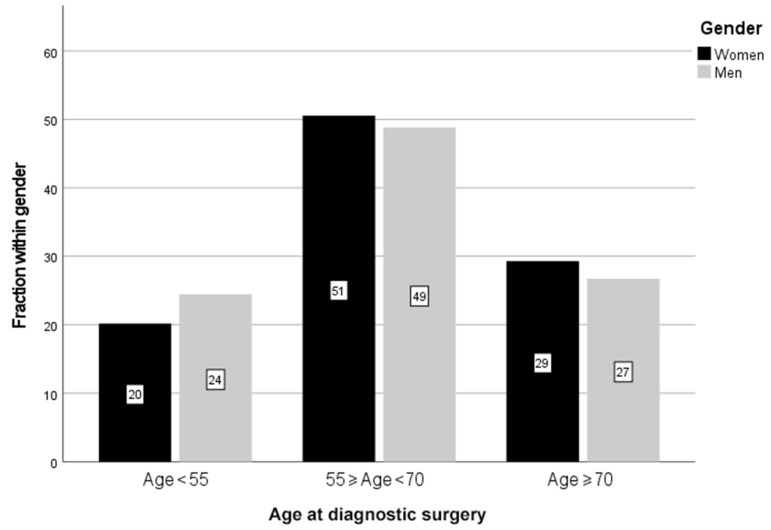
Age at the time of diagnostic surgery for men and women. Only 20% of all women were diagnosed below the age of 55 years, while for men this was 24%. A larger proportion of women were diagnosed in the age groups ≥55 & <70 years and ≥70 years than for the men (*p* = 0.001), using the Chi square test. The faction within each sex is reported in percent (%).

A total of 5243 patients with the pathological diagnosis of GBM, giant cell GBM, or gliosarcoma were reported to SQRBT between 1 January 1999 and 31 December 2018. This means that approximately 90% of all patients diagnosed with GBM in Sweden were reported to the quality registry from all six regions every year, apart from two regions that did not report during 2000–2005 and 2005–2011, respectively. This was calculated compared to the number of patients reported to the Swedish Cancer Registry, where all cancer cases in Sweden are registered. The vast majority, 96.3%, were GBM (SNOMED 94403), with gliosarcoma and giant cell GBM constituting only 3.7% together. Of all patients, 2083 were women and 3160 men, giving a ratio of 1:1.5. The median age at the time of diagnostic surgery was higher in women than in men, namely 64 versus 63 years, and with only 1 in 5 being diagnosed below the age of 55 for women, in contrast to near one quarter in men (*p* = 0.001) (Table 1 and Figure 1). 

**Figure 2:**
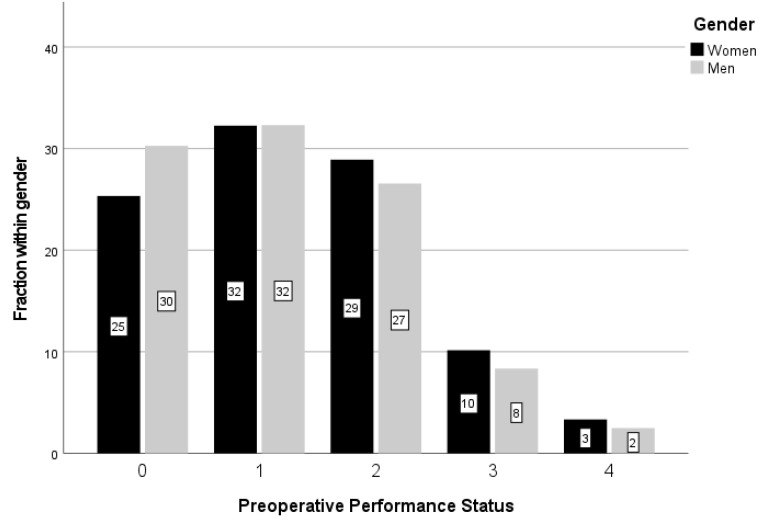
WHO preoperative performance status (PPS) for men and women. Significantly more men had a better PPS (*p* < 0.001) using the Chi square test. The fraction within each sex is reported in percent (%).

Interestingly, WHO PPS was judged to be 0 in considerably more men than women, and conversely, less men were reported with PPS 2, 3 and 4, with these sex differences being significant (*p* < 0.001) (Figure 2).

We found no significant differences for the type of surgery performed in males and females during the investigated period, with 26.7% of males having a biopsy versus 26.3% of females. The corresponding percentages for partial resection were 40.1% and 39.3%, and for radical resection 33.3% and 34.4% (*p* = 0.71). 

**Figure 3:**
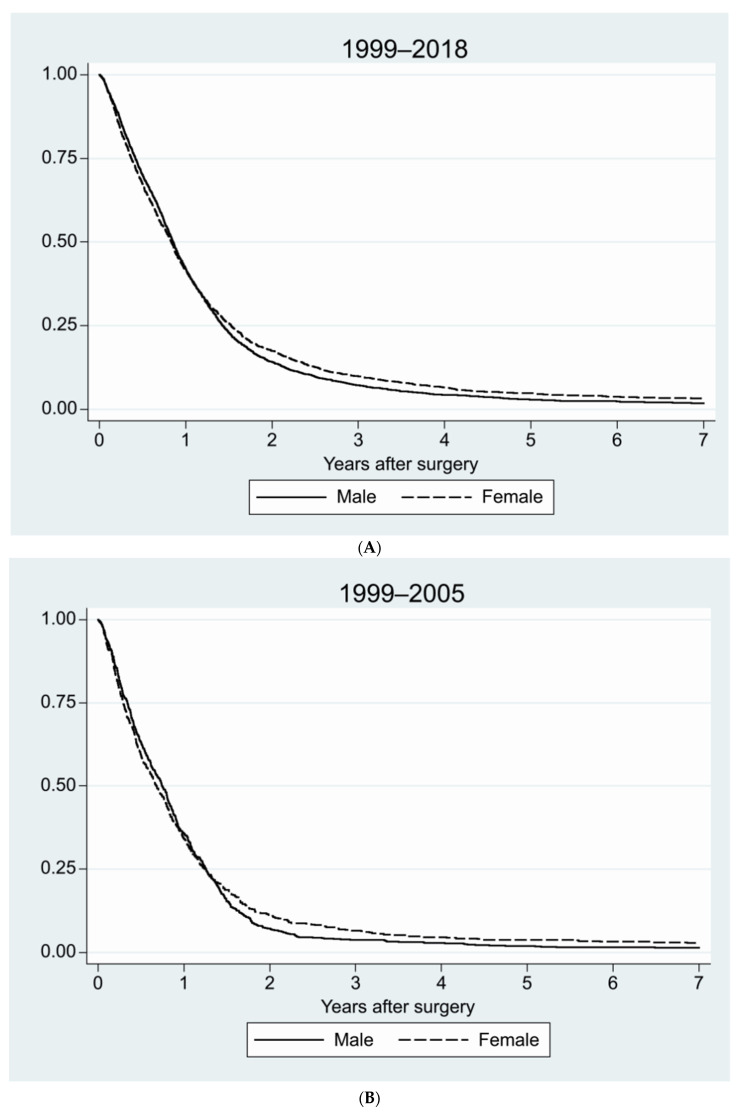

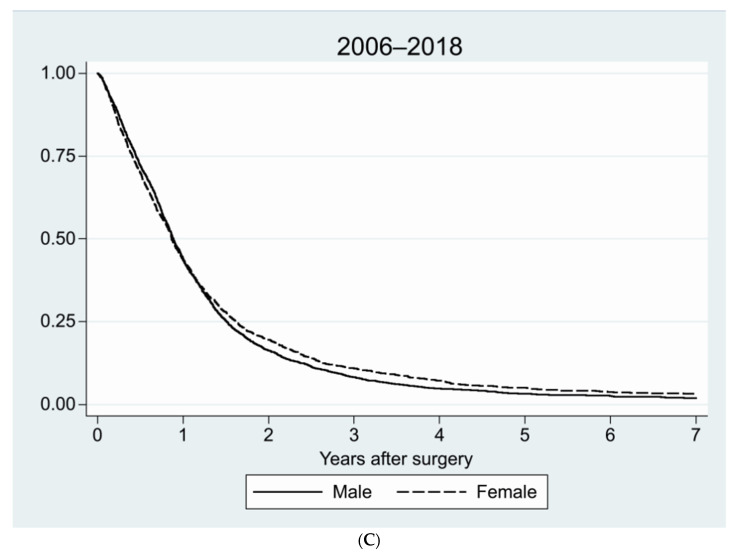
Survival for men and women for different time periods. (**A**) For the complete 20-year period from 1999–2018 (*p* = 0.06); (**B**) 1999–2005, before temozolomide was introduced into routine treatment (*p* = 0.35); (**C**) 2006–2018, after the introduction of concomitant radio-chemotherapy with temozolomide (*p* = 0.10), using Kaplan–Meier survival curves.

We compared survival for men versus women for the whole 20-year period, and for the periods 1999–2005 and 2006–2018. For the 20-year period, the median survival was 312 days (95%CI 301–323 days) for men and 305 days for women (290–320 days), this difference being near significant (*p* = 0.06). Median survival for the earlier period was 271 days for men (95%CI 247–295 days) and 248 for women (95%CI 215–281 days) (*p* = 0.35). For the later period, survival had increased in general, but still no differences were noted related to sex, with the median survival for men being 324 days (95%CI 311–337 days) and for women 316 days (95%CI 299–333 days) (*p* = 0.10) (Figure 3A–C).

**Figure 4:**
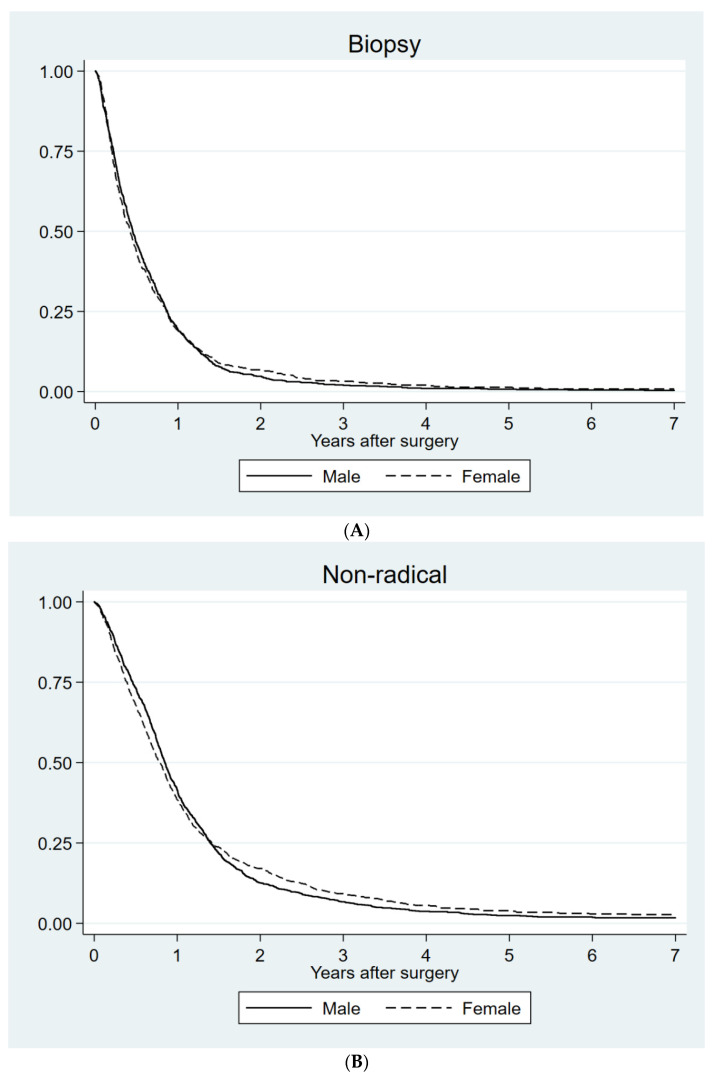

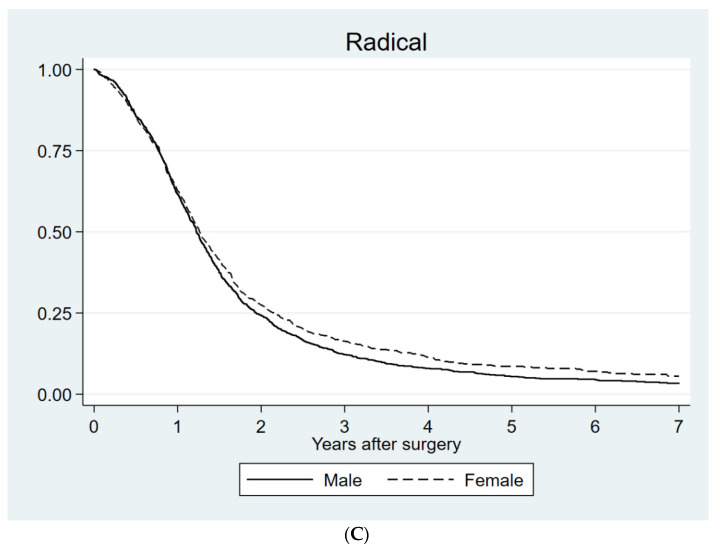
Survival for men and women with different types of diagnostic surgery. (**A**) Biopsy (*p* = 0.86); (**B**) partial resection (*p* = 0.66); (**C**) radical resection (*p* = 0.045) using Kaplan–Meier survival curves.

We also searched for potential differences in survival between sexes related to the type of surgery. As expected, the best survival was found for those who had undergone radical surgery and the poorest was for those biopsied only. For women with radical resection, a small but statistically significant survival advantage was noted (*p* = 0.045) (Table 2 and Figure 4A–C).

An additional multivariate analysis, including the prognostic factors age, PPS, type of surgery, and sex, revealed a statistically significant survival advantage for women with HR 0.92 (95%CI 0.87–0.98; *p* = 0.006; Table 3). 

Lastly, the role of lead times was investigated. The median time from radiologic diagnosis to surgery was 17 days for men and 18 days for women, and from surgery to the pathology report it was 8 days for all patients.

## 4. Discussion

For decades it has been known that GBM affects far more men than women. In the last 10 years, there has been an increasing interest in investigating the factors that could cause this disparity and how these can influence outcomes. Whether, in line with personalized medicine, these factors could or should lead to different tumor treatments according to the sex of the patient is a hot topic [20,21]. 

Several publications have addressed the sex disparity in GBM incidence. An interesting study investigating the discrepancy in GBM incidence between men and women found that intracranial volume (ICV), as a substitute for brain size, was correlated to the risk of developing high-grade glioma, with increasing ICV leading to a higher risk [22]. This was believed to be associated with a larger number of neuroglial stem-cell divisions in a larger brain, and could be one factor accounting for a larger proportion of GBM in men. Sun et al. found that loss of p53 function increases the risk of malignant transformation, in particular in male astrocytes, being an additional mechanism for the differences in incidences [23]. The p53-associated sex disparity is also observed in other cancers, which is linked to the location of negative regulators of p53 on the X chromosomes [24]. A further study found that the genetic risk for acquiring glioma was associated with EGFR in males, and to TERT instead in females, indicating biologically relevant sex differences in gliomagenesis [25]. 

A debated topic without a definitive answer today is the role of hormones [26,27,28,29,30]. Interestingly, we did note a small but significant difference related to age at diagnostic surgery, with female patients generally being older, which could suggest a role for hormonal factors. Estrogen is found at higher concentrations in female brains, where it binds to nuclear or membrane receptors. This leads to many different and complex effects that could contribute to the sex disparity [26]. While some studies have found a correlation of glioma incidence to exogenous hormone treatment, such as oral contraceptives or hormone replacement therapy, others have not [28,30]. Regarding the findings in GBM tumor tissue, a high expression of prolactin and the prolactin receptor has been shown to have a detrimental effect on survival in males only [29]. Interestingly, the androgen receptor (AR) gene has been found to be amplified in both male and female GBM, and AR RNA was overexpressed in more than 90% of the samples examined [31].

Yang et al. found a better tumor response to standard treatment in females, detected by imaging, and even sex differences at the transcriptome level, with cell cycle signaling being of importance for male survival and integrin signaling for females [8]. In the study by Johansen et al., different methylation patterns were identified for males and females with IDHwt GBM [7]. This would lead to differentially expressed genes. Additional molecular markers have been reported to be differentially expressed in GBM, depending on the sex of the patient. Such a marker is the WNT-receptor Frizzled-7 [32], where high Frizzled was correlated to shorter survival in men only. In the same study, surprisingly, the methylated MGMT promoter was not a significant factor for survival for male patients treated with alkylating agents. We, together with others, have reported that a larger proportion of women with GBM have MGMT promoter methylated tumors, approximately 50%, in comparison to only around one third in men [10,11,12,19]. Apart from this, our data seem to support women having a better response to alkylating agent treatment when the MGMT promoter is methylated. On the other hand, women treated with temozolomide are more likely to be affected by serious hematological toxicity [33]. 

An unexpected finding in our cohort was that women, in general, were found to have worse PPS compared to men, the reason for this being unclear. This is a factor related to the judgment of the neurosurgeon. Despite its subjectivity, PPS has been shown to be an important prognostic factor for patients with GBM [34], as we found in our multivariate analysis. 

In our population-based dataset, we noted an increase in survival over time in the total cohort of GBM patients, this surely mainly reflecting the introduction of concomitant radio-chemotherapy, in line with previous reports [1,34]. During the later time period, additional improvements in the treatment of GBM patients could also have contributed [35], including the use of 5-ALA during surgery [36].

In the present study, including the vast majority of all GBM patients diagnosed in Sweden between 1999 and 2018, we could not detect any survival difference between men and women in the cohort in general, although there was a trend towards better survival for men. As both age and PPS, where we noted differences related to sex, are important prognostic factors that will have an impact on the patient´s survival together with the type of surgery, a multivariate analysis including these factors was done. This showed a small survival advantage for women, although this was not clinically relevant. In addition, in the subgroup of those with radical resection, a small female survival benefit of just 14 days was found in median. This could be argued to be in line with previous publications in this area, describing better survival for women [7,12,19]. On the other hand, a recently published report from the CBTRUS noted a small but significant survival disadvantage for female patients with GBM, with HR 1.02 (*p* < 0.001), as compared to men [37]. One explanation could be, as indicated above, that different biological factors, some surely yet to be identified, have a different or opposite effect on survival for men and women. Other reasons could be the oncological tumor treatment provided to the patients, a factor that was not available for this analysis, this constituting a shortcoming of the study. If only a portion of all patients were fit enough to receive concomitant radio-chemotherapy, a survival difference that would have been expected to favor females with MGMT promoter methylated tumors, who received temozolomide treatment, could go undetected. In fact, data from Norway, a similar health care setting to Sweden, imply that only approximately 43% of patients diagnosed with GBM will be candidates for concomitant and adjuvant radio-chemotherapy [38]. This could then also possibly be an explanation for the small survival benefit that was noted for the subgroup of women with radical resection, as patients with radical surgery are more likely to receive more extensive oncological treatment such as concomitant radio-chemotherapy with temozolomide. 

The treatment related factors investigated in this study, namely lead times and type of surgery performed, were the same among men and women, pointing towards equal care regardless of sex for patients with GBM in Sweden.

Another limitation of this study is that molecular markers were not available. As mentioned above, approximately 10% of this cohort could be expected to be IDHmut GBM (now astrocytoma grade 4) [5,18]. As this is a new entity, the role of sex is still not completely known from larger datasets. According to the CBTRUS report, anaplastic astrocytoma (now astrocytoma grade 3) (A3) were more frequent in males, with no significant survival difference between males and females [37]. In line with findings from the CATNON trial, also in the CBTRUS data, the majority of A3 patients were surely IDHmut [16]. We note that the CBTRUS findings for A3 are similar to that of IDHwt GBM, with male dominance, but with small differences in survival between sexes, if any. This in turn indicates that the inclusion of IDHmut GBM (astrocytoma grade 4) in the Swedish cohort should not have a major impact on the results reported here. 

## 5. Conclusions

In this population-based dataset of Swedish patients diagnosed with GBM during a 20-year period, we identified that the prognostic factors of age at the time of diagnostic surgery and preoperative performance status differ according to sex, apart from the well-known difference in incidence between men and women. Despite this, survival was not clearly affected for men or women when focusing on the total cohort. For the subgroup of radically resected patients, a minor survival difference favoring women was identified. 

For future cohorts, diagnostic molecular markers will be known, thus allowing for correct selection of IDHwt GBM. Even data on oncological treatment will be available. This will undoubtedly further contribute to the understanding of the role of sex for prognosis and the effect of tumor specific therapy in GBM in a population-based cohort. 

Additional study of the molecular disparities and their effect on outcome can be expected to lead to improved sex-dependent tumor treatments in the future. This might lead to increased survival for both men and women, and then also on a population level, as the incorporation of concomitant radio-chemotherapy with temozolomide did more than a decade ago.

## Figures and Tables

**Table 1:** Age at diagnostic surgery according to sex for patients with GBM between 1999–2018. Percentages reflect the fraction for each sex separately.

Age, Years	Sex	Number	Percent, %
18–39	Men	131	4.1
	Women	70	3.4
40–54	Men	642	20.3
	Women	350	16.8
55–69	Men	1543	48.8
	Women	1053	50.6
≥70	Men	844	26.7
	Women	610	29.3

**Table 2:** Median survival in relation to sex and type of surgery. Better survival was noted for more extensive surgery, with a sex difference for radical resection favoring women. Significant *p*-value in bold.

	Sex	Median Survival, Days	95% Confidence Interval, Days	*p*-Value
Biopsy	Men	165	149–181	0.86
	Women	153	132–174	
Partial resection	Men	311	296–326	0.66
	Women	291	269–313	
Radical resection	Men	447	424–470	**0.045**
	Women	461	431–491	

**Table 3:** Multivariate analysis including the prognostic factors; preoperative performance status (PPS), age, type of surgery, and sex.

Prognostic Factor		Hazard Ratio	95% Confidence Interval	Significance, *p*-Value
Preoperative Performance status (WHO)	0–1	1.00		
	2	1.27	1.19–1.36	<0.001
	3–4	1.89	1.72–2.07	<0.001
Age	≤54	1.00		
	55–69	1.59	1.47–1.71	<0.001
	≥70	2.46	2.26–2.68	<0.001
Surgery	Radical	1.00		
	Partial	1.47	1.37–1.57	<0.001
	Biopsy	2.43	2.25–2.62	<0.001
Sex	Male	1.00		
	Female	0.92	0.87–0.98	0.006

## Data Availability

The data presented in this study are available upon request from the corresponding author. The data are not publicly available due to the database being continually expanded with additional cases and only data relevant for this analysis were retrieved.
